# Reduction in Antibiotic Prescribing Attainable With a Gonococcal Vaccine

**DOI:** 10.1093/cid/ciab276

**Published:** 2021-03-31

**Authors:** Stephen M Kissler, Moriah Mitchell, Yonatan H Grad

**Affiliations:** 1 Department of Immunology and Infectious Diseases, Harvard T. H. Chan School of Public Health, Boston, Massachusetts, USA; 2 Department of Epidemiology, Harvard T. H. Chan School of Public Health, Boston, Massachusetts, USA

**Keywords:** gonorrhea, vaccine, antibiotic prescribing, antibiotic resistance

## Abstract

We estimated the fraction of antibiotic prescribing in the United States attributable to gonorrhea. Gonorrhea contributes to an outsized proportion of antibiotic prescriptions in young adults, males, and in the southern and western United States. A gonococcal vaccine could substantially reduce antibiotic prescribing in these populations.

Antibiotic resistance poses a growing threat to human health [1]. Antibiotic resistance is driven by the consumption of antibiotics, which exert evolutionary selective pressures on the intended target pathogen and on “bystander” micro-organisms [2]. Vaccination can reduce the incidence of illnesses that prompt antibiotic prescriptions, thereby reducing downstream antibiotic resistance [3].

Gonorrhea is the second most prevalent reportable illness in the United States, with nearly 600 000 cases reported in 2018 [4], and is a major contributor to morbidity worldwide [5]. Until December 2020, the US Centers for Disease Control and Prevention (CDC) recommended treating gonorrhea with oral azithromycin plus intramuscular ceftriaxone. Now, in response to rising rates of azithromycin resistance, monotherapy with intramuscular ceftriaxone is recommended [6]. Due to its high prevalence, gonorrhea contributes substantially to antibiotic prescribing.

There are currently no licensed gonococcal vaccines, although modest protection has been demonstrated from the MeNZB *Neisseria meningitidis* vaccine and a clinical trial of meningococcal group B vaccine (Bexsero) for gonorrhea is ongoing [7, 8]. An effective gonococcal vaccine could profoundly reduce gonorrhea-associated morbidity. However, the extent of impact of a gonococcal vaccine on antibiotic prescribing has not been quantified. Here, we integrated reported cases of gonorrhea in the United States with a large database capturing outpatient antibiotic prescriptions to estimate the amount of prescribing attributable to gonorrhea and the reduction in antibiotic prescribing that would be possible with a gonococcal vaccine.

## METHODS

### Study Sample and Population

We extracted annual cases of gonorrhea and chlamydia by state, sex, and age group (0–14, 15–19, 20–24, 25–29, 30–34, 35–39, 40–44, 45–54, 55–64, ≥65 years) in the United States from 2015 through 2018, the most recent years available, from the CDC’s *AtlasPlus* portal [9]. We used the population sizes for each demographic and geographic group, available from the same source, to calculate cases per 1000 individuals (Table 1).

**Table 1.** Gonorrhea Cases and Antibiotic-Prescribing Rates per 1000 Persons in the United States

		Azithromycin		Ceftriaxone		Total Antibiotics	
	Cases/1000 Persons	Rx/1000 Persons (Overall)	Percent Due to Gonorrhea	Rx/1000 Persons (Overall)	Percent Due to Gonorrhea	Rx/1000 Persons (Overall)	Percent Due to Gonorrhea
Year							
2015	1.23	148	0.83	1.6	78.2	728	0.34
2016	1.45	142	1.02	1.8	79.8	730	0.40
2017	1.70	131	1.30	2.1	81.6	706	0.48
2018	1.79	119	1.50	2.2	82.9	680	0.53
Age (2018) (years)							
** **0–14	0.05	87	0.06	0.2	20.2	762	0.01
** **15–19	4.32	130	3.32	4.8	89.6	712	1.21
** **20–24	7.12	137	5.21	7.7	93.0	598	2.38
** **25–29	5.53	115	4.83	6.0	92.3	565	1.96
** **30–34	3.66	120	3.06	4.2	87.7	621	1.18
** **35–39	2.28	126	1.81	2.7	85.8	658	0.69
** **40–44	1.37	125	1.09	1.8	76.8	653	0.42
** **45–54	0.74	127	0.58	1.0	72.8	665	0.22
** **55–64	0.29	136	0.21	0.6	44.6	739	0.08
** ≥**65	0.05	171	0.03	3.7	1.2	797	0.01
Sex (2018)							
Female	1.46	140	1.04	1.9	79.0	806	0.36
Male	2.13	97	2.19	2.5	86.0	549	0.78
HHS region (2018)							
** **1 (Boston)	1.07	96	1.11	2.0	55.0	585	0.37
** **2 (New York City)	1.60	126	1.27	2.0	81.6	714	0.45
** **3 (Washington, DC)	1.46	112	1.31	2.0	73.2	677	0.43
** **4 (Atlanta)	2.06	139	1.48	2.5	83.9	769	0.54
** **5 (Chicago)	1.81	109	1.65	2.2	83.4	654	0.55
** **6 (Dallas)	1.92	145	1.32	2.3	85.6	779	0.49
** **7 (Kansas City)	1.98	103	1.93	2.6	76.2	635	0.62
** **8 (Denver)	1.37	86	1.58	2.7	50.0	553	0.49
** **9 (San Francisco)	1.96	103	1.89	2.2	90.8	546	0.72
** **10 (Seattle)	1.46	72	2.04	2.2	65.6	479	0.61

Abbreviations: HHS, US Department of Health and Human Services; Rx, prescriptions.

We extracted outpatient antibiotic prescriptions from the Truven MarketScan database [10] over the same time frame. This database captures all outpatient pharmacy claims from a convenience sample of 19.1–24.3 million individuals (5.9–7.6% of the US population), depending on the month (Supplementary Table 1). We linked each antibiotic prescription with the patient’s age, sex, and state. Full extraction details are given in the Supplementary Methods.

Next, we estimated the number and proportion of azithromycin prescriptions, ceftriaxone prescriptions, and overall antibiotic prescriptions in the United States that were attributable to gonorrhea in 2015–2018. Treatment for gonorrhea is normally administered under the supervision of a provider upon diagnosis, so these antibiotics are not recorded in the outpatient prescriptions data. We therefore added 1 dose of azithromycin and 1 dose of ceftriaxone for each reported gonorrhea infection. Similarly, we added 1 dose of azithromycin for each reported case of chlamydia. This is a conservative choice, since chlamydia may be treated by a single supervised dose of azithromycin (recommended) or a 7-day course of doxycycline. Full details on these adjustments are given in the Supplementary Methods, including a sensitivity analysis accounting for deviations from the recommended gonorrhea treatment regimen (Supplementary Table 2).

For the years 2015–2018, we calculated the percentage and total number of azithromycin prescriptions, ceftriaxone prescriptions, and total antibiotic prescriptions per 1000 people that were attributable to gonorrhea. For 2018, when gonorrhea incidence was at its highest level in over a decade, we calculated the same statistics stratified by age, sex, and US Department of Health and Human Services (HHS) region [11]. Vaccine-induced reductions in prescribing were estimated using the product of vaccine uptake and efficacy within plausible ranges. Full details are given in the Supplementary Methods.

## RESULTS

There were 1.79 reported cases of gonorrhea per 1000 individuals in the United States in 2018. These accounted for 1.5% of all azithromycin prescriptions, 82.9% of all ceftriaxone prescriptions, and 0.53% of all antibiotic prescriptions of any type given in 2018 (Table 1). The incidence of gonorrhea rose by a factor of 1.5 between 2015 and 2018. Meanwhile, overall antibiotic-prescribing rates fell, so that the proportion of antibiotic prescriptions due to gonorrhea increased faster than the increase in gonorrhea incidence alone would suggest. Gonorrhea cases were mainly concentrated in young adults. There were 7.12 cases of gonorrhea reported per 1000 individuals between the ages of 20 and 24 years in 2018, accounting for 2.38% of all antibiotic prescriptions in that age group. Gonorrhea cases were less frequent in females than in males (1.46 vs 2.13 cases per 1000 individuals in 2018, respectively). Gonorrhea cases in 2018 were lowest in the Northeast (1.07 cases per 1000 people in HHS region 1, Boston) and highest in the Southeast (2.06 cases per 1000 people in HHS region 4, Atlanta). However, gonorrhea-related antibiotic prescriptions accounted for the greatest share of overall antibiotic prescriptions in the West (0.72% of all antibiotic prescriptions in HHS region 9, San Francisco) due to a relatively high rate of gonorrhea infections and a relatively low rate of antibiotic prescribing for other conditions [12].

The updated ceftriaxone monotherapy treatment guidelines for gonorrhea will effectively halve the total number of antibiotic prescriptions due to gonorrhea. Gonorrhea-associated azithromycin prescribing will decline effectively to zero, while gonorrhea-associated ceftriaxone prescribing should remain roughly unchanged, barring major changes in the relative prevalence of gonorrhea and other illnesses treatable by ceftriaxone.

We illustrate the potential impact of a gonococcal vaccine on antibiotic prescribing by assuming vaccine uptake of 50% (*u* = 0.5), consistent with adolescent human papillomavirus (HPV) vaccine uptake in the United States [13], and by considering a modest vaccine efficacy of 50% (*e* = 0.5) and a high vaccine efficacy of 90% (*e* = 0.9). In 2018, a gonococcal vaccine with 50% uptake and 50% efficacy would have reduced gonorrhea-related antibiotic prescribing by an estimated 25%, so that the overall percentage of antibiotic prescriptions due to gonorrhea would have been 0.40%. Among 20- to 24-year-olds, such a vaccine would have reduced the proportion of antibiotic prescriptions due to gonorrhea from 2.38% to 1.78%. For a vaccine with 50% uptake and 90% efficacy, gonorrhea-related prescribing would decline by an estimated 45% and thus gonorrhea would have accounted for 0.29% of all antibiotic prescriptions and 1.31% of antibiotic prescriptions among 20- to 24-year-olds. Had monotherapy been in place in 2018, the baseline proportion of antibiotic prescriptions due to gonorrhea (Table 1, final column) would have been half as large. A vaccine with 50% uptake and 90% efficacy would have reduced the proportion of antibiotics due to gonorrhea by an additional factor of 0.45. In total, the estimated percentage of antibiotic prescriptions due to gonorrhea would have been just 0.14%, a 72.5% reduction from the actual value. Overall, a gonococcal vaccine is likely to have the greatest impact on prescribing where gonorrhea-associated prescribing is highest, including in young adults, males, and the southern and western United States.

## DISCUSSION

We have estimated the share of antibiotic prescribing in the United States attributable to gonorrhea infections. Gonorrhea incidence and associated gonorrhea-related antibiotic prescribing are especially variable between age groups, with the highest incidence and highest associated prescribing rates in young adults. Since overall antibiotic-prescribing rates are lowest in this same age group, a gonococcal vaccine would yield an outsized benefit in this age group in terms of the fraction of antibiotic prescriptions averted. Reducing antibiotic prescribing in demographic groups that otherwise receive few antibiotics may be an especially effective strategy for combatting antibiotic resistance, since a given antibiotic course is expected to contribute more to resistance in an individual who receives few other prescriptions than in an individual who already receives many [14]. Taken together, these findings suggest that a gonococcal vaccine could lead to a substantial reduction in antibiotic prescribing, helping to combat the rise in antibiotic resistance.

Gonorrhea accounts for a majority of outpatient ceftriaxone prescriptions, which will likely remain unchanged in the switch to monotherapy. Ceftriaxone may also be used to treat community-acquired pneumonia [15] and otitis media [16], among numerous other indications, and its use may also reflect post-hospitalization treatment courses for endovascular, musculoskeletal, and other infections. The bacteria that cause many of these conditions—such as *Streptococcus pneumoniae* and *Escherichia coli*—can be carried commensally and therefore may be subject to bystander evolutionary selection pressures [2]. As rates of gonorrhea increase, ceftriaxone resistance should be closely monitored in these other pathogens.

Our findings align with others that have documented higher rates of gonorrhea in the southern United States, in males, and in young adults [4]. Similarly, elevated rates of antibiotic prescribing in the southern United States and in children and older adults have been documented [17]. Gonorrhea incidence has rapidly risen over the past decade. We anticipate that this trend will continue, making gonorrhea account for an even larger share of antibiotic prescriptions and increasing the potential benefit of a gonococcal vaccine for reducing both gonorrhea-related morbidity and associated antibiotic prescribing. Furthermore, a gonococcal vaccine could reduce rates of unspecified urethritis and pelvic inflammatory disease, which also prompt antibiotic prescriptions.

While we have focused on vaccination, other measures to reduce gonorrhea incidence, such as increased condom use and changes in treatment regimen, could also lead to reductions in antibiotic prescribing. We only consider the direct effects of vaccination (ie, blocking symptomatic disease in the vaccine recipient); if a vaccine also reduces gonococcal transmission, further reductions in prescribing may be expected. We have focused on gonorrhea incidence in the United States, but we anticipate that a gonococcal vaccine would similarly lead to substantial reductions in antibiotic prescribing worldwide.

## Supplementary Data

Supplementary materials are available at *Clinical Infectious Diseases* online. Consisting of data provided by the authors to benefit the reader, the posted materials are not copyedited and are the sole responsibility of the authors, so questions or comments should be addressed to the corresponding author.

ciab276_suppl_Supplementary_Material

## Notes

***Financial support.*** This work was supported by funding from the Wellcome Trust.

***Potential conflicts of interest.*** Y. H. G. has received consulting income from Quidel, Merck, and GSK; research support form Merck and Pfizer; and is on the scientific advisory board of Day Zero Diagnostics. All other authors report no potential conflicts. All authors have submitted the ICMJE Form for Disclosure of Potential Conflicts of Interest. Conflicts that the editors consider relevant to the content of the manuscript have been disclosed.
