# Supplementary material for: Reduction in Antibiotic Prescribing Attainable With a Gonococcal Vaccine
Source: Clin Infect Dis. 2021 Mar 31;73(6):e1368–71. doi: 10.1093/cid/ciab276 (PMC8522794; doi:10.1093/cid/ciab276)
Supplement: ciab276_suppl_Supplementary_Material [file ciab276_suppl_supplementary_material.docx]

**Supplement to:**

Reduction in antibiotic prescribing attainable with a gonococcal vaccine

Stephen M. Kissler^1^*, Moriah Mitchell^2^*, Yonatan H. Grad^1^

^1^Department of Immunology and Infectious Diseases, Harvard. T.H. Chan School of Public Health, Boston MA, USA

^2^Department of Epidemiology, Harvard. T.H. Chan School of Public Health, Boston MA, USA

* contributed equally

Correspondence: Yonatan H. Grad ([ygrad@hsph.harvard.edu](mailto:ygrad@hsph.harvard.edu))

Alternate corresponding author: Stephen Kissler ([skissler@hsph.harvard.edu](mailto:skissler@hsph.harvard.edu))

**Supplemental methods**

Extracting antibiotic claims from the MarketScan database. To extract antibiotic prescribing rates from the MarketScan database, we began by extracting the age, sex, state of enrollment, and member identification number for every person with a full month of insurance enrollment for each month between January 2015 and December 2018. **Supplemental Table 1** lists the number of members enrolled in each month and the proportion of the US population represented. For each member in each month, we then extracted all associated antibiotic claims. Antibiotics were identified by the National Drug Code (NDC) number using a lookup file containing the therapeutic class and drug name provided by Truven MarketScan. To estimate the annual *per capita* antibiotic prescribing rate, we divided the total number of antibiotics prescribed in a given year by the total number of member-months in that year (*i.e.,* the sum of the number of members enrolled across all months in that year) and multiplied by 12. Multiplying this quantity by 1,000 yielded the number of antibiotic prescriptions per 1,000 people in that year. We performed these extractions for azithromycin prescriptions, ceftriaxone prescriptions, and all antibiotic prescriptions.

Adjusting antibiotic prescribing rates for reported cases of gonorrhea and chlamydia. The recommended course of azithromycin and ceftriaxone for treating gonorrhea is normally administered under the supervision of a provider upon diagnosis, so these prescriptions are not recorded in the MarketScan outpatient prescriptions data. We therefore added one dose of azithromycin and one dose of ceftriaxone for each reported gonorrhea infection to the outpatient antibiotic prescriptions data extracted from MarketScan. We also accounted for antibiotic prescriptions for chlamydia, the most common reportable illness in the US, by adding one dose of azithromycin to the MarketScan outpatient prescriptions data for each reported chlamydia case. This is a conservative choice, since chlamydia may be treated by a single supervised dose of azithromycin (recommended) or a 7-day course of doxycycline. By assuming that all cases of chlamydia prompt an azithromycin dose, this will lead to an over-estimate of the number of azithromycin prescriptions that are actually given, yielding more conservative estimates of the relative impact of a gonococcal vaccine on azithromycin prescribing.

Accounting for deviations from recommended treatment course. An estimated 4-10% of gonorrhea cases in New York City in 2011-13 were treated with monotherapy rather than dual therapy^18^. To account for reduced antibiotic prescribing due to monotherapy treatment, we conducted a conservative supplemental analysis in which each documented case of gonorrhea generated 0.9 azithromycin prescription and 1 ceftriaxone prescription. This assumes that the upper estimate of 10% of gonorrhea cases were treated with ceftriaxone monotherapy (the current CDC recommendation) rather than ceftriaxone and azithromycin dual therapy. The revised figures are reported in **Supplemental Table 2.** The change yields minor adjustments in the percent of azithromycin and overall antibiotic prescriptions due to gonorrhea.

Quantifying the reduction in prescribing due to vaccination. The potential reduction in antibiotic prescribing due to a gonococcal vaccine can be estimated as a function of vaccine uptake and efficacy. The expected proportion of gonorrhea cases directly prevented by vaccination is *x = u*e*, where *u* is the proportion of the population that has been vaccinated and *e* is the vaccine efficacy, *i.e.,* the proportion of illnesses prevented by vaccination. If *p* is the proportion of all antibiotic prescriptions due to gonorrhea in the absence of vaccination, then the estimated proportion of antibiotic prescriptions due to gonorrhea with vaccination is *p_v_ = p * (1-x).*

| **Year** | **Month** | **Members (millions)** | **Percent of US population** |
| --- | --- | --- | --- |
| 2015 | 1 | 24.3 | 7.61 |
|  | 2 | 24.3 | 7.60 |
|  | 3 | 24.3 | 7.58 |
|  | 4 | 24.2 | 7.56 |
|  | 5 | 24.1 | 7.54 |
|  | 6 | 24.1 | 7.51 |
|  | 7 | 24.0 | 7.50 |
|  | 8 | 24.0 | 7.46 |
|  | 9 | 23.9 | 7.45 |
|  | 10 | 23.9 | 7.44 |
|  | 11 | 23.8 | 7.42 |
|  | 12 | 23.8 | 7.39 |
| 2016 | 1 | 24.1 | 7.49 |
|  | 2 | 24.1 | 7.49 |
|  | 3 | 24.1 | 7.48 |
|  | 4 | 24.1 | 7.47 |
|  | 5 | 23.9 | 7.42 |
|  | 6 | 24.0 | 7.42 |
|  | 7 | 23.9 | 7.41 |
|  | 8 | 23.9 | 7.38 |
|  | 9 | 23.8 | 7.36 |
|  | 10 | 23.7 | 7.33 |
|  | 11 | 23.6 | 7.30 |
|  | 12 | 23.5 | 7.27 |
| 2017 | 1 | 19.8 | 6.12 |
|  | 2 | 19.8 | 6.11 |
|  | 3 | 19.8 | 6.10 |
|  | 4 | 19.7 | 6.08 |
|  | 5 | 19.7 | 6.06 |
|  | 6 | 19.6 | 6.05 |
|  | 7 | 19.6 | 6.02 |
|  | 8 | 19.5 | 6.01 |
|  | 9 | 19.5 | 5.99 |
|  | 10 | 19.5 | 5.98 |
|  | 11 | 19.2 | 5.91 |
|  | 12 | 19.2 | 5.89 |
| 2018 | 1 | 21.1 | 6.48 |
|  | 2 | 21.1 | 6.48 |
|  | 3 | 21.1 | 6.46 |
|  | 4 | 21.0 | 6.45 |
|  | 5 | 21.0 | 6.43 |
|  | 6 | 20.9 | 6.41 |
|  | 7 | 20.9 | 6.39 |
|  | 8 | 20.9 | 6.38 |
|  | 9 | 20.8 | 6.37 |
|  | 10 | 20.7 | 6.34 |
|  | 11 | 20.7 | 6.33 |
|  | 12 | 20.6 | 6.30 |

**Supplemental Table 1. Number of Insurance members represented in the MarketScan database.** The percent of the US population represented in the MarketScan data is calculated using the monthly resident population of the United States as reported by the United States Census Bureau (https://www.census.gov/data/tables/time-series/demo/popest/2010s-national-total.html)


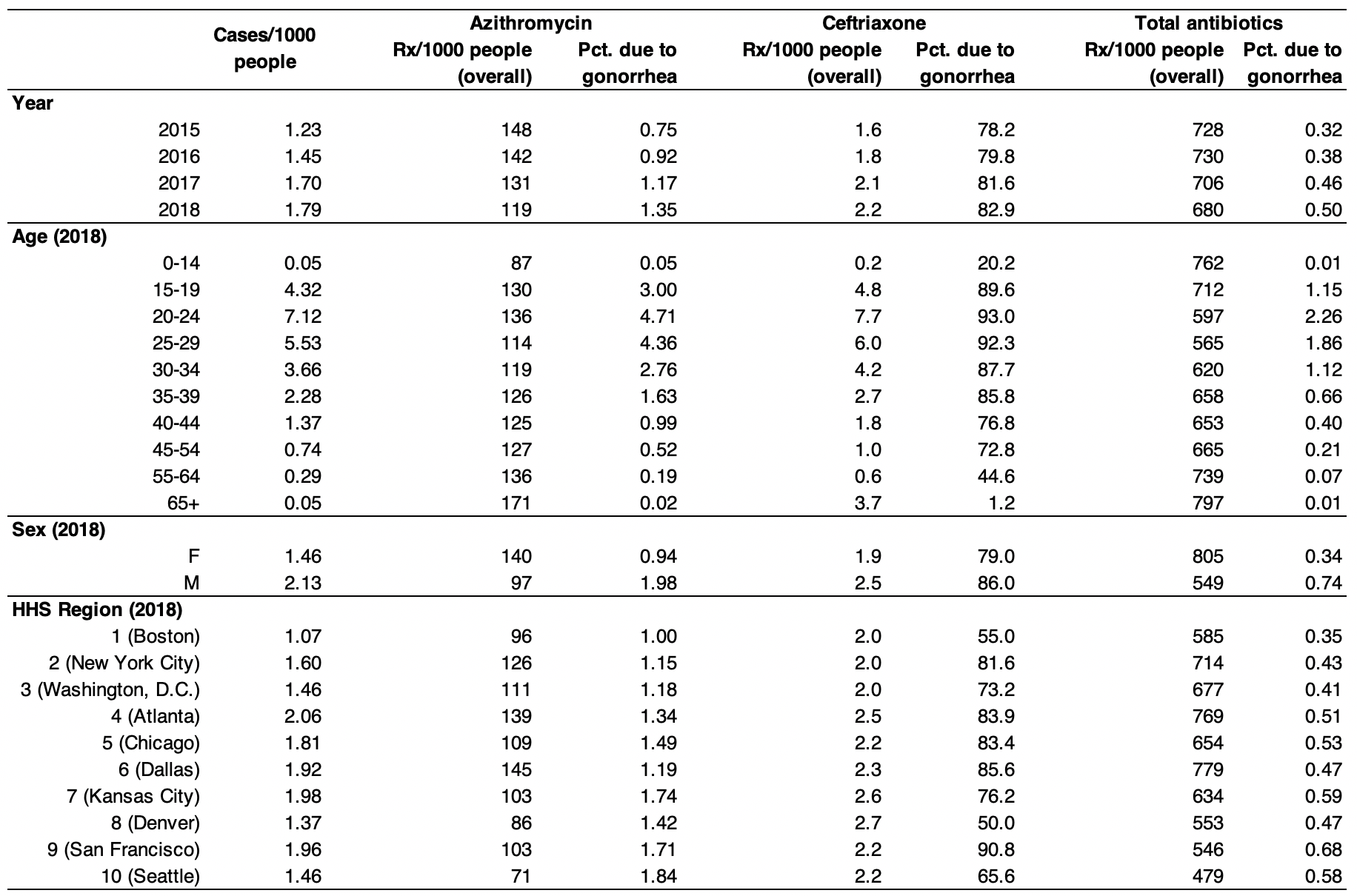


**Supplemental Table 2. Gonorrhea cases and antibiotic prescribing rates per 1,000 people in the United States accounting for deviation from treatment recommendations.** An estimated 4-10% of gonorrhea infections were treated with monotherapy rather than the recommended dual therapy. To account for this, we conservatively assumed that each case of gonorrhea generated 0.9 azithromycin prescription and 1 ceftriaxone prescription. Rx=prescriptions; pct=percent.
